# 系统性鉴定长非编码RNA MALAT1调控的微小RNA

**DOI:** 10.3779/j.issn.1009-3419.2016.05.11

**Published:** 2016-05-20

**Authors:** 海天 张, 国祥 王, 荣 尹, 满堂 邱, 林 许

**Affiliations:** 1 221004 徐州，徐州医科大学临床医学系 Department of Clinical Medicine, Xuzhou Medical University, Xuzhou 221004, China; 2 221000 徐州，徐州医科大学附属医院心胸外科 Department of Cardiothoracic Surgery, Affiliated Hospital of Xuzhou Medical University, Xuzhou 221000, China; 3 210009 南京，南京医科大学附属江苏省肿瘤医院胸外科/江苏省恶性肿瘤分子生物学及转化医学重点实验室 Department of Thoracic Surgery, Nanjing Medical University Affiliated Cancer Hospital, Jiangsu Key Laboratory of Molecular and Translational Cancer Research, Cancer Institute of Jiangsu Province, Nanjing 210009, China

**Keywords:** 长非编码RNA, MALAT1, microRNA, 生物信息学, Long non-coding RNA, MALAT1, MicroRNAs, Bioinformatics

## Abstract

**背景与目的:**

长非编码RNA（long non-coding RNA, lncRNA）在肿瘤的发生、侵袭转移等过程中发挥着重要的调控作用。本研究通过实验和生物信息学手段系统性研究lncRNA MALAT1调控的微小RNA（microRNA, miRNA）。

**方法:**

设计特异性敲减MALAT1的反义寡核苷酸（antisense oligonucleotides, ASO），在A549细胞中敲减MALAT1，通过TqaMan Low Density Array（TLDA）芯片研究敲减MALAT1后miRNA表达变化；使用基因集富集分析（gene set enrichment analysis, GSEA）方法分析敲减MALAT1之后差异表达基因，寻找富集的miRNA。

**结果:**

ASO有效降低了MALAT1表达，敲减MALAT1之后153个miRNA表达显著变化，其中131个miRNA表达上调，22个miRNA表达下调。在A549细胞中敲减MALAT1后，458个基因发生显著差异表达，GSEA分析发现多个miRNA在差异表达基因中被显著富集。对TLDA和GSEA数据取交集并进一步分析确认28个被MALAT1调控的miRNA。

**结论:**

本研究系统鉴定了MALAT1对miRNA的调控，为进一步研究提供了基础。

## Abstract (en)

**Background and objective:**

Long non-coding RNA (lncRNA) plays important regulatory roles in the development and invasion of various cancers. The aim of current study is to comprehensively identify microRNAs (miRNA) regulated by lncRNA MALAT1 via experimental and bioinformatics methods.

**Methods:**

Antisense oligonucleotides (ASO) specifically targeting MALAT1 were designed and synthesized. After knockdown of MALAT1 by ASO in A549 cells, miRNA expression changes were profiled by TqaMan Low Density Array (TLDA). Gene set enrichment analysis (GSEA) was used to search enriched miRNAs among differentially expressed genes after knockdown of MALAT1.

**Results:**

After efficient knockdown of MALAT1 by ASO, 153 miRNAs were differentially expressed, 131 up-regulated and 22 down-regulated. Among the 458 differentially expressed genes after MALAT1 silence, GSEA results revealed lots of enriched miRNAs. There were 28 overlapped miRNAs between TLDA and GSEA results, suggesting these 28 miRNAs are regulated by MALAT1.

**Conclusion:**

This study comprehensively identified MALAT1 regulated miRNAs, providing resources for further research.

长非编码RNA（long non-coding RNA, lncRNA）广泛参与了各种生理和疾病过程，在肺癌的发生和进展过程中也发挥着重要的调控作用^[1, 2]^。MALAT1是肺癌中最早被鉴定的lncRNA之一，也是肺癌研究领域中研究最多的lncRNA之一，很多研究都证实MALAT1可以促进肺癌的恶性进展^[3-5]^。

MALAT1在肺癌中的分子机制目前已有相关报道，但是对MALAT1与微小RNA（microRNA, miRNA）的相互调控关系的研究尚少^[6, 7]^。此外目前关于MALAT1与miRNA的研究多是集中在miRNA对MALAT1表达的负性调控^[7]^，而MALAT1对miRNA表达的调控作用尚未见系统性研究。因此，本研究旨在通过实验和生物信息手段，系统性地研究MALAT1调控的miRNA。

## 1 材料与方法

### 1.1 实验材料

A549细胞购买于中国科学院上海细胞库，H1640培养基购于南京凯基公司，RNA提取试剂Trizol购自Invitrogen公司，逆转录试剂盒和实时定量PCR试剂盒购于Takara公司，TaqMan Low Density Array（TLDA）芯片购于AppliedBiosystems公司，转染试剂Lipo2000购于Invitrogen公司。实验所用引物和反义寡核苷酸（antisense oligonucleotides, ASO）于南京金斯瑞公司合成，MALAT1引物序列：上游5’-GGATCCTAGACCAGCATGCC-3’，下游5’-AAAGGTTACCATAAGTAAGTTCCAGAAAA-3’；β-肌动蛋白引物序列上游：5’-GAAATCGTGCGTGACATTAA-3’，下游：5’-AAGGAAGGCTGGAAGAGTG-3’。ASO序列：ASO1：5’-ATGGAGGTATGACATATAAT-3’，ASO2：5’-TCTTATGTTTCCGAACCGTT-3’ ^[3, 4]^。

### 1.2 细胞培养与转染

A549细胞培养在10%FBS的H1640培养基中，37 ℃、5%CO_2_饱和湿度培养。将A549细胞接种于六孔板中转染，使用Lipo2000试剂以终浓度100 nmol/L转染ASO^[4, 8]^。RT-PCR法检测MALAT1表达：A549细胞转染24 h后使用Trizol法提取总RNA，根据试剂盒说明进行逆转录反应。RT-PCR反应体系：cDNA 0.5 μL、水3.7 μL、上下游引物各0.4 μL、2×reaction mix 5 μL，使用ABI 7900仪器进行RT-PCR反应，每组实验均以β-actin作为内参^[9, 10]^。

### 1.3 TaqMan Low Density Array（TLDA）实验

A549细胞转染后提取总RNA并逆转录，使用ABI 7900仪器根据制造商说明书进行TLDA芯实验，使用制造商提供的RQ manage软件进行数据分析。

### 1.4 基因集富集分析（Gene Set Enrichment Analysis, GSEA）

将差异表达基因按照差异倍数从高到低排序，使用GSEA软件中的GseaPreranked选项进行数据分析，miRNA基因集注释文件下载自GSEA网站^[11]^。

### 1.5 统计学方法

采用SPSS 18.0软件完成统计分析。使用Student’s t检验计算MALAT1表达差异，*P* < 0.05为差异有统计学意义。

## 2 结果

### 2.1 在A549细胞中敲减MALAT1

在A549细胞中转染诊断MALAT1的ASO序列和阴性对照序列（scramble），与对照组相比，转染ASO1和ASO2后MALAT1表达被下调，且ASO1效果更佳（图 1A）。

**1:**
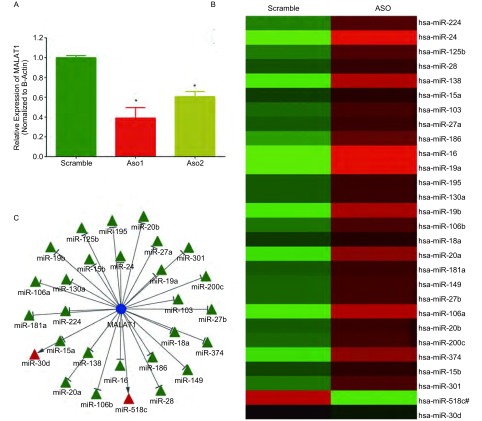
相对于阴性对照序列（scramble），ASO1和ASO2抑制了MALAT1表达水平，ASO1下调作用更佳，^*^P < 0.05（A）；28个miRNA在TLDA芯片中表达水平，红色表达上调，绿色表达下调（B）；MALAT1和miRNA调控网络，蓝色：MALAT1，绿色：敲减MALAT1后表达上调miRNA，红色：敲减MALAT1后表达下调的miRNA（C） ASO1 and ASO2 significantly inhibited MALAT1 expression level, compared with scramble sequence, and ASO1 showed better inhibitory effect. ^*^P < 0.05 (A). Expression level of the 28 miRNAs in TLDA results, red: up-regulation, green: down-regulation (B); regulatory network of MALAT1 and miRNAs; blue: MALAT1, green: miRNAs up-regulated after MALAT1 knockdown, red: miRNAs down-regualted after MALAT1 knockdown

### 2.2 TLDA实验

使用ASO1在A549细胞中敲减MALAT1，通过TLDA实验检测miRNA表达变化，以*P* < 0.05为筛选标准，共发现131个上调、22个下调的miRNA。

### 2.3 GSEA分析

GutschnerT在A549细胞中敲减MALAT1后通过基因表达谱芯片发现458个差异表达基因^[3]^，本研究对这些差异表达基因进行GSEA分析，发现有多个miRNA在这些差异表达基因中被显著富集。

### 2.4 MALAT1调控的miRNA

将TLDA得到的差异表达miRNA和GSEA富集的miRNA取交集，获得45个miRNA，进一步筛选表达与调控关系相符的miRNA，最终获得28个被MALAT1调控的miRNA（表 1，图 1B）。根据MALAT1与这些miRNA的表达关系，我们构建了MALAT1-miRNA调控网络（图 1C）。

**1:** 28个MALAT1调控的miRNA 28 miRNAs regulated by MALAT1

Name	GSEA	TLDA regulation	TLDA fold change
miR-186	neg	up	14.065
miR-24	neg	up	12.942
miR-125b	neg	up	15.295
miR-18a	neg	up	6.850
miR-130a	neg	up	13.539
miR-138	neg	up	13.614
miR-28	neg	up	13.520
miR-224	neg	up	13.242
miR-19a	neg	up	29.878
miR-374	neg	up	14.065
miR-15a	neg	up	6.625
miR-27a	neg	up	13.492
miR-181a	neg	up	12.888
miR-149	neg	up	13.436
miR-103	neg	up	14.430
miR-106a	neg	up	13.671
miR-15b	neg	up	6.718
miR-106b	neg	up	13.871
miR-195	neg	up	13.482
miR-20b	neg	up	13.699
miR-16	neg	up	12.545
miR-19b	neg	up	12.746
miR-200c	neg	up	14.153
miR-20a	neg	up	14.133
miR-27b	neg	up	13.728
miR-301	neg	up	26.119
miR-518c	pos	down	-6.038
miR-30d	pos	down	-2.213
pos: positively enriched by GSEA; neg: negatively enriched by GSEA; up: up-regulated after MALAT1 knockdown; down: down-regualted after MALAT1 knockdown.			

## 3 讨论

MALAT1是2004年Ji等^[12]^发现的、一个长8, 556核苷酸的反义lncRNA，MALAT1位于11号染色体。MALAT1在肺癌组织中显著高表达，且MALAT1高表达提示患者预后不良^[13]^，同时体内和体外实验都证实MALAT1可以促进肺癌细胞侵袭和增殖能力^[1, 14]^。在食管癌、胃癌、肝癌等其他肿瘤中，MALAT1也发挥着促癌基因作用^[15-17]^。

关于MALAT1的分子生物学机制已有较多研究，但是对于MALAT1调控miRNA方面研究较少。本研究首先设计并合成了特异性靶向MALAT1的ASO，并有效的敲减了MALAT1的表达。通过TLDA芯片，发现敲减MALAT1之后很多miRNA表达发生显著变化，证实MALAT1会影响miRNA表达，提示miRNA可能通过调控miRNA表达来发挥生物学功能。Gutschner等^[3]^通过ZFN技术敲减了MALAT1，并发现了458个差异表达基因，我们使用GSEA方法分析了这些差异表达基因，寻找这些基因中被富集的miRNA结合位点。GSEA可以分析一个预先定义的基因集是否被富集^[9]^。敲减MALAT1后miRNA表达下调，则miRNA靶基因表达上调；对差异表达基因GSEA分析，miRNA会被正性富集。因此，在对TLDA和GSEA取交集的45个miRNA中进一步筛选出下调-正性富集和上调-负性富集的28miRNA，即这28个miRNA是被MALAT1调控的miRNA。

Tripathi等^[4]^首先报道MALAT1可以影响RNA剪切，而后Gutschner等^[3]^通过芯片分析发现MALAT1敲减之后对RNA剪切影响不大，而对基因表达水平有重要影响，提示MALAT1可以调控基因转录。之后，诸多研究^[17, 18]^发现MALAT1可以通过与RNA结合蛋白绑定调控下游基因的转录。因此MALTA1也可能通过与RNA结合蛋白绑定直接调控miRNA转录；此外，受MALAT1调控的基因也可能间接调控miRNA表达。然而，对于本研究鉴定出的28个miRNA，还需进一步实验来明确MALAT1具体是通过何种机制调控这些miRNA的表达。

本研究通过实验和生物信息学分析手段、系统性地研究了MALTAT1调控的miRNA，为进一步研究MALAT1和miRNA的调控网络提供了参考。

## References

[1] Qiu MT, Hu JW, Yin R (2013). Long noncoding RNA: an emerging paradigm of cancer research. Tumor Biol.

[2] Esteller M (2011). Non-coding RNAs in human disease. Nat Rev Genet.

[3] Gutschner T, Hammerle M, Eissmann M (2013). The noncoding RNA MALAT1 is a critical regulator of the metastasis phenotype of lung cancer cells. Cancer Res.

[4] Tripathi V, Ellis JD, Shen Z (2010). The nuclear-retained noncoding RNA MALAT1 regulates alternative splicing by modulating SR splicing factor phosphorylation. Mol Cell.

[5] Zhou X, Liu S, Cai G (2015). Long non coding RNA MALAT1 promotes tumor growth and metastasis by inducing epithelial-mesenchymal transition in oral squamous cell carcinoma. Sci Rep.

[6] Han X, Yang F, Cao H (2015). Malat1 regulates serum response factor through miR-133 as a competing endogenous RNA in myogenesis. FASEB J.

[7] Wang X, Li M, Wang Z (2015). Silencing of long noncoding RNA MALAT1 by miR-101 and miR-217 inhibits proliferation, migration, and invasion of esophageal squamous cell carcinoma cells. J Biol Chem.

[8] Jiang M, Wang Q, Karasawa T (2014). Sodium-glucose transporter-2 (SGLT2; SLC5A2) enhances cellular uptake of aminoglycosides. PLoS One.

[9] Qiu M, Xu Y, Wang J (2015). A novel lncRNA, LUADT1, promotes lung adenocarcinoma proliferation via the epigenetic suppression of p27. Cell Death Dis.

[10] Jiang M, Zhang C, Wang J (2011). Adenosine A(2A)R modulates cardiovascular function by activating ERK1/2 signal in the rostral ventrolateral medulla of acute myocardial ischemic rats. Life Sci.

[11] Subramanian A, Tamayo P, Mootha VK (2005). Gene set enrichment analysis: a knowledge-based approach for interpreting genome-wide expression profiles. Proc Natl Acad Sci U S A.

[12] Ji P, Diederichs S, Wang W (2003). MALAT-1, a novel noncoding RNA, and thymosin beta4 predict metastasis and survival in early-stage non-small cell lung cancer. Oncogene.

[13] Pang EJ, Yang R, Fu XB (2015). Overexpression of long non-coding RNA MALAT1 is correlated with clinical progression and unfavorable prognosis in pancreatic cancer. Tumour Biol.

[14] Schmidt LH, Spieker T, Koschmieder S (2011). The long noncoding MALAT-1 RNA indicates a poor prognosis in non-small cell lung cancer and induces migration and tumor growth. J Thorac Oncol.

[15] Li C, Chen J, Zhang K (2015). Progress and prospects of long noncoding RNAs (lncRNAs) in hepatocellular carcinoma. Cell Physiol Biochem.

[16] Li S, Wang Q, Qiang Q (2015). Sp1-mediated transcriptional regulation of MALAT1 plays a critical role in tumor. J Cancer Res Clin Oncol.

[17] Hirata H, Hinoda Y, Shahryari V (2015). Long noncoding RNA MALAT1 promotes aggressive renal cell carcinoma through Ezh2 and interacts with miR-205. Cancer Res.

[18] Ji Q, Zhang L, Liu X (2014). Long non-coding RNA MALAT1 promotes tumour growth and metastasis in colorectal cancer through binding to SFPQ and releasing oncogene PTBP2 from SFPQ/PTBP2 complex. Br J Cancer.

